# Validation of WINROP algorithm as screening tool of retinopathy of prematurity among Egyptian preterm neonates

**DOI:** 10.1038/s41433-024-02953-1

**Published:** 2024-02-03

**Authors:** Asmaa Fares, Sherif Abdelmonaim, Dina Sayed, Sherin Sadek, Abdulrahman Abdulrazek, Youssef Helmy, Sara Maher

**Affiliations:** 1 Pediatrics Department, Cairo University, Cairo, Egypt; 2 El Galaa Teaching Hospital, The general organization of teaching hospitals and institutes, Cairo, Egypt; 3 Ophthalmology Department, Fayoum University, Cairo, Egypt; 4 Ophthalmology Department, Cairo University, Cairo, Egypt

**Keywords:** Outcomes research, Retinal diseases

## Abstract

**Background:**

Retinopathy of prematurity (ROP) is a leading cause of preventable childhood blindness worldwide. Proper screening for ROP can prevent loss of vision. WINROP (weight, insulin-like growth factor 1, neonatal, retinopathy of prematurity) is an online surveillance system based on gestational age, birth weight and weekly weight gain that can predict infants at risk of sight-threatening retinopathy of prematurity.

**Aims:**

To evaluate the diagnostic accuracy of WINROP algorithm in detecting sight-threatening ROP in Egyptian preterm neonates.

**Methods:**

Birth weight (BW), gestational age (GA) and weekly weight measurement of 365 preterm infants were prospectively entered into WINROP algorithm. Based on these inputs, the algorithm would output and a screening was performed as is standard. Sensitivity, specificity, and predictive values were calculated by comparing WINROP outcomes with ROP screening outcomes.

**Results:**

Of the infants included in the study the mean GA was ±31.24 and mean BW was ±1508.78. A high risk WINROP alarm was triggered in 62 infants of whom 16 infants develop type 1 or type 2 ROP. These infants had associated comorbidities including sepsis, Intraventricular hemorrhage (IVH), Necrotizing enterocolitis (NEC), history of transfusion of packed red blood cells (RBCS) and history of platelet transfusion. A low risk WINROP alarm was triggered in 303 infants of whom 15 infants developed type 1 or type 2ROP. WINROP showed a sensitivity of 51.6%, a specificity of 86.2%, a positive predictive value (PPV) of 52.8% and a negative predictive value (NPV) of 95% for detection of type 1 or type 2 ROP.

**Conclusion:**

WINROP has low sensitivity and high specificity for detection of ROP. It may help in ROP prediction but can’t be used alone. Modification of WINROP algorithm taking into account other risk factors may improve sensitivity and reduce number for ROP examination.

## Introduction

Retinopathy of prematurity (ROP) is a vasoproliferative disease that affects the developing retinal vascular system in premature infants. It is a leading causes of preventable childhood blindness worldwide and continues to be a challenge in neonatology [1].

Prevalence of ROP range from 7% to 37% in different regions of the world. It ranges from 10% to 27% in developed countries depending on degree of prematurity and birth weight [2].

In Egypt, there are approximately 1.88 million live births every year, of whom 136,000 are born prematurely. Currently there is no nationwide standardized ROP screening program. The prevalence of ROP in Egypt ranges from 19.2 to 69.4% [3].

The major risk factors for ROP development are earlier gestational age, low birth weight, and high concentration oxygen therapy. The incidence and course of ROP are thought to be influenced by other risk factors, including anemia, sepsis, postnatal weight gain, serum levels of insulin-like growth factor-1 (IGF-1), thrombocytopenia, bilirubin level, gender, multiple gestations, intraventricular hemorrhage, and blood transfusion [4].

The international classification of ROP has provided clinicians and researchers with a common language to describe ROP. ROP is divided into 5 stages based on its progression. The retina is divided into 3 circular zones centered on the optic disc. The calibre and tortuosity of retinal blood vessels are also described. A diagnosis of ROP involves specifying the stage of the disease, the zone of vascular termination and the presence or absence of dilated and tortuous blood vessels (plus disease) [5].

WINROP (weight, insulin-like growth factor 1 (IGF-1), neonatal retinopathy of prematurity risk algorithm) (www.winrop.com) is an online-based surveillance system that was developed to use the rate of weight gain in the first 4 weeks after birth as a substitute for serum levels of IGF-1 to predict the occurrence of severe ROP. This System has the potential to reduce the number of infants who require screening [6].

## Patients and methods

This is a prospective cohort study that was carried out in the neonatal intensive care units of Cairo University Hospitals (Kasr Al Ainy) and the legislation Association Hospitals for premature infants and newborns from June 2020 to December 2021. The University Hospital Research Committee approved the study protocol. The study and data collection conformed to all local laws and were compliant with the principles of Declaration of Helsinki. Informed consent was taken from guardians of neonates. Inclusion criteria: preterm neonates with gestational age (GA) ≤ 34 weeks and/or birth weight (BW) ≤ 2000 g. Exclusion criteria: Infants with retinal diseases other than ROP, non-physiological weight gain as in hydrocephalus, incomplete weight measurements and infants who did not have complete retinopathy screening.

For every infant included the following data was recorded: gestational age (GA) calculated based on the first day of last menstrual period, birth weight (BW), weekly weight, gender, method of conception (Natural, Intracytoplasmic sperm injection or In Vitro Fertilization), method of delivery (Normal Vaginal or Caesarian section), number of births in pregnancy (Single, Twins, Triplets or more), maternal illness and neonatal comorbidities (sepsis, necrotizing enterocolitis, intraventricular hemorrhage, patent ductus arteriosus, need for blood or blood products transfusion, respiratory support, length of hospital stay). Weekly weight was input into the WINROP system until 36 weeks post conceptual age or until the WINROP system provided an alarm. The WINROP algorithm compares each infant’s weekly weight gain to an expected weight curve. An alarm was triggered if the sum of the deviation was greater than a predetermined limit, indicating that the infant was at high risk of developing ROP that would need treatment. The WINROP outcomes were an alarm or no alarm which represented a high or low risk for developing type 1 ROP, respectively.

All infants included had ROP screening as is standard; infants were first examined at the 4th week of life or at 31 weeks of post conceptual age (whichever was later) by a pediatric ophthalmologist with experience in ROP screening using binocular indirect Ophthalmoscopy. Severity of ROP was graded according to The Early treatment of ROP study (ETROP) into Type 1 ROP (zone I, any stage with plus disease or zone I stage 3 ROP without plus disease, or zone II, stage 2 or 3 with plus disease), Type 2 ROP (zone I, stage 1 or 2 without plus disease, or zone II, stage 3 without plus disease) and No ROP. ROP requiring treatment was type 1 ROP or type 2 ROP with signs of progression according to the Early Treatment Retinopathy of Prematurity study (ETROP). Subsequent exams were performed weekly or at a wider interval, according to the findings of the last ophthalmic examination. Screening was terminated when the retina was completely vascularized or blood vessels were within 1 disc diameter of the ora serrata in infants with no prior ROP. With infants who had ROP, screening continued until complete regression and zone III vascularization. Finally, the results of WINROP were compared with the Results of ROP screening.

### Statistical analysis

Quantitative parametric data were presented as mean, standard deviations ranges. Quantitative non-parametric data were presented as median and inter-quartile range (IQR). Qualitative data were presented as number and percentages.

Quantitative data between 2 groups were compared using the Independent t-test one (parametric) and the Mann–Whitney test (Non-parametric). Quantitative data between more than 2 groups were compared using the One-Way ANOVA test (parametric) and the Kruskall–Wallis test. (Non-parametric).

### The comparison between groups with qualitative data was done using Chi-square test

Receiver operating characteristic curve (ROC) was used in the qualitative form to determine sensitivity, specificity, positive predictive value (PPV), negative predictive value (NPV) and accuracy for the studied parameters to predict ROP of the studied patients. The sample size was calculated with an assumption that the prevalence of ROP was 5% and sensitivity of WINROP algorithm was 95% with a 10% margin of error and a 95% confidence interval. This led to a sample size of 365 infants.

## Results

This study included 365 infants. 48.2% were female (*n* = 176). The mean gestational age was 31.24 weeks ±1.33, while mean birth weight was 1508.78 g ± 301. Infants who were born with a gestational age > 32 weeks were included if their birth weight was <1500 g. Similarly, infants whose birth weight was >1500 g were included if their gestational age was <32 weeks.

287 infants were delivered by caesarian section while the remaining 78 infants were delivered by normal vaginal delivery. As regards neonatal comorbidities, 56.4% had sepsis, 40.3% received packed RBCS transfusion 22.2% had intraventricular hemorrhage (IVH), 12.1% had a patent ductus arteriosus (PDA), and 6.0% had Necrotizing enterocolitis (NEC). The median days of respiratory support were 10 (4–20) days, mean days of hospital stay were 42.85 ± 19.22 days and a high risk WINROP alarm was activated a median of 2 weeks (1–3).

Among the 365 infants included in the study, 4.7% (*n* = 17) developed type 1 ROP, 3.8% (*n* = 14) developed type 2 ROP and 91.5% (*n* = 334) did not develop ROP.A WINROP High risk alarm was triggered in 17% (*n* = 62) of the infants, while low risk alarm was triggered in 83% (*n* = 303) infants.

The 17 patients who developed type 1 showed a mean GA of 29.24 weeks ±1.86 (27–32) and a mean BW of 1444.12 ± 309.46 (900–2000).

There was a statistically significant correlation between a high risk WINROP alarm and a diagnosis of type 1 ROP and type 2 ROP (*p* = 0.007, 0.000 respectively), and similarly, between a low risk WINROP alarm a diagnosis of no ROP (*p* = 0.000) (Table 1).

**Table 1:** Comparison between WINROP results with results of fundus examination.

	WINROP results				*P*-value
	High risk		Low risk		
	No = 62	%	No = 303	%	
ROP					
No ROP	46	74.2%	288	95.0%	0.000
ROP type 1	7	11.3%	10	3.3%	0.007
ROP type 2	9	14.5%	5	1.7%	0.000

Among the 62 infants who had a high-risk alarm, only 16 actually developed ROP. Conversely among the 288 infants who had a low-risk alarm, 15 infants eventually developed ROP. This means that the WINROP algorithm gave a false positive alarm in 30 patients and a false negative in 15 patients.

Accordingly, the sensitivity of WINROP was 51.6%, the specificity was 86.2%, the positive predictive value was 25.8% and the negative predictive value was 95% (Table 2).

**Table 2:** Predictive performance in detecting ROP using the WINROP algorithm.

	No ROP		ROP (Type1 or 2)	
	*n*	%	*n*	%
Low Risk WINROP alarm	288	86.2%	15	48.4%
High Risk WINROP alarm	46	13.8%	16	51.6%

There was a statistically significant correlation between a high-risk WINROP alarm and low gestational age (*p* < 0.0001), low birth weight (*p* < 0.0001), sepsis (*p* < 0.0001), intraventricular hemorrhage (*p* < 0.0001), necrotizing enterocolitis (*p* = 0.013), packed RBCS transfusion (*p* < 0.0001), platelet transfusion (*p* = 0.001), days of respiratory support (*p* < 0.0001) and days of hospital stay in the studied neonates (*p* < 0.0001).

## Discussion

The incidence of ROP varies in different regions of the world. In one study it was found to range from 7 to 37%. This variability in incidence was attributed to the variability in average prevalent gestational age and birth weight of preterm infants in different countries around the world [2].

The severity of ROP also varies with more severe ROP seen in low and middle-income countries [7]. In our study, conducted on 365 preterm infants, in 2 Egyptian neonatal units, the incidence of ROP was 8.5%.

As neonatal services improve worldwide, the incidence of ROP is expected to increase. The gold-standard to prevent ROP- related infant blindness is screening with indirect ophthalmoscopy. In a world with finite resources, an ideal screening program needs to be efficient to avoid overloading the healthcare system and subjecting infants who would not get ROP to unnecessary examination, without missing infants at risk [7].

Several ROP prediction models have been developed to predict high risk patients in an attempt to reduce the frequency of unneeded examinations and to improve ROP screening efficiency without missing cases with severe forms of the disease that would need treatment [8].

Many of these models are based on the correlation between treatment-requiring ROP and low serum levels of Insulin-like growth factor, and the concomitant correlation between slow postnatal weight gain and low serum levels of insulin-like growth factor. Thus, establishing a correlation between slow postnatal weight gain and treatment-requiring ROP [9].

Among these models that use the rate of postnatal weight gain to predict worse ROP are the WINROP algorithm (weight, insulin-like growth factor 1 (IGF-1), neonatal retinopathy of prematurity risk algorithm), the Children’s Hospital of Philadelphia postnatal weight gain, birth weight (BW), and gestational age (GA) ROP risk model(CHOP-ROP), the Colorado-retinopathy of prematurity model (Co-ROP), The Digital ROP model (DIGIROP), The Postnatal Growth and ROP study model (G-ROP) and The Alexandria retinopathy of prematurity model (Alex-ROP) [10].

These models have been found to reduce the number of examinations by 25–75% while maintaining acceptable sensitivity for predicting severe ROP [11].

In our study we attempted to validate the sensitivity and specificity of the WINROP model on a population of Egyptian preterm infants.

The WINROP model was developed in Sweden. In the original validation study conducted on 50 preterm infants in a Swedish neonatal unit, it showed a sensitivity of 100% and a specificity of 54% for detecting severe ROP. The infants included had a range of gestational age between 23.0 to 30.6 weeks and birth weight between 460–1716 g. In their study, they defined sensitivity as the probability that an alarm is called for an infant who is actually at risk and specificity as the probability that an alarm is not called for an infant who is not risk [6].

In our study we demonstrated a much less sensitivity at 51.6% and a higher specificity of 86.2%.

Multiple studies have validated The WINROP algorithm in different populations and showed varying degrees of sensitivity and specificity [12].

Looking at the varying results we are able to note that these studies were conducted in different countries with different levels of income and on infants of different ethnicities They are summarized in Table 3 [12–30].

**Table 3:** Results of WINROP algorithm in different studies.

Studies	Country	Number	Sensitivity (%)	Specificity (%)
Hellstrom et al. [14]	Sweden	353	100.0	84.5
Hard et al. [15]	Brazil	366	90.5	55.0
Wu et al. [16]	USA and Canada	1706	98.6	38.7
Zepeda-Romero et al. [17]	Mexico	352	84.7	26.6
Ko et al. [18]	Taiwan	148	64.7	–
Piermarocchi et al. [19]	Italy	377	83.6	55.2
Kocak et al. [20]	Turkey	223	84.3	52.8
Timkovic et al. [21]	Czech Republic	445	100	69.7
Jung et al. [22]	Canada	483	81.8	53.3
Sanghi et al. [23]	India	70	90.32	38.46
Ueda et al. [24]	Japan	278	84.4	42.7
Lim et al. [8]	Malaysia	92	95.2	33.8
Raffa et al. [25]	Saudi Arabia	175	100	31.5
Chaves-Samaniego et al. [26]	Spain	502	76.1	74.8
Bai et al. [27]	China	432	56	–
Desai et al. [28]	Australia	221	85.7	59
Fernández-Ramón et al. [29]	Spain	109	100	55.9
Sute et al. [13]	India	153	80	80.6
Almeida et al. [30]	Portugal	311	87.1	56

Varying results in the literature (and by inference on our population) could be due to a variety of factors, including differences in preterm study populations, study design, inclusion criteria, different epidemiology of ROP in different countries due to different standards for neonatal care and exposure to risk factors, and a lower survival rate of extremely preterm infants due to prenatal and postnatal care limitations. Furthermore, the so-called “normal predicted weight gain curve” can vary per country [27].

A meta-analysis of 36 studies (*n* = 11,500) conducted by Athikarisamy et al. found that the WINROP algorithm had a sensitivity of 89% (95 percent CI, 0.85–0.92) and a specificity of 57% in predicting type 1 or severe ROP (95 percent CI, 0.51–0.63). High-Income vs. Low- to Middle-Income Countries had different WINROP sensitivity and specificity. The sensitivity was 91% (95 percent CI, 0.85–0.95) and the specificity was 60% (95 percent CI, 0.53–0.66) in 24 studies conducted in high-income countries (*n* = 8543), while the sensitivity was 85% (95 percent CI, 0.78–0.90) and the specificity was 51% in 12 studies conducted in low- to middle-income countries (*n* = 2957) (95 percent CI, 0.39–0.64) [31].

One possible unique factor in our cohort of patients- that may have influenced our results-was the relatively older and heavier infants included. Our inclusion criteria were infants who had a GA ≤ 32 weeks or a BW ≤ 1500 g. This meant that infants were included if they fulfilled one of these criteria even if they did not fulfill the other. Looking at our results, the mean gestational age was 31.2 weeks with a standard deviation (SD) of ±1.33 weeks and a range of 26–32 weeks. Despite not including infants >32 weeks of GA, the mean and SD show that at least two thirds of the included infants had GA 28.5 weeks or more and so had relatively older GA when compared to the original WINROP validation study. A closer look at BW shows a mean of 1508.78 g with a standard deviation of 301.11 grams and a range of 690–2400 g. Here it is clear that preterm infants with BW > 1500 g were included and two thirds of the infants were heavier than 900 g, once again demonstrating that our study included heavier infants.

The reason these infants were included is that they are normally included in our ROP screening based on fulfilling one criterion for screening and we wanted our results to reflect our real-world practice.

The subgroup analysis of the infants who developed treatment-requiring ROP showed a mean GA of 29.24 weeks ±1.86 (27–32) and a mean BW of 1444.12 ± 309.46 (900–2000). This demonstrates that some infants with a BW > 1500 g (and up to 2000g) do develop treatment-requiring ROP. This justifies including them in our study, despite the possibility of that inclusion having skewed our results.

In Conclusion. When applying the WINROP model to our population of infants, it appears more accurate in correctly predicting infants who were not at risk for ROP, rather than the opposite. Based on our results, we do not recommend using The WINROP algorithm instead of standard screening.

Post hoc analysis of infants with BW < 1500 g in our study population or a prospective study excluding infants with BW < 1500 g may demonstrate different sensitivity and specificity and may inform on further value for the use of WINROP in Egyptian neonatal units.

## Summary

### What was known before

- WINROP algorithm is a useful screening tool for ROP.

### What this study adds

- Validation of WINROP algorithm as a screening tool for ROP among Egyptian preterm neonates.

## Data Availability

The data that support the findings of this study are available from the corresponding author upon reasonable request.

## References

[1] Enrı´quez AB, Avery RL, Baumal CR (2020). Update on Anti-Vascular Endothelial Growth Factor Safety for Retinopathy of Prematurity. Asia Pac J Ophthalmol.

[2] Bhuiyan ANH, Mannan MA, Dey SK, Choudhury N, Shameem M, Shahidullah M (2019). Frequency and Risk Factors for Retinopathy of Prematurity in Very Low Birth Weight Infants in NICU, BSMMU. TAJ: J Teach Assoc.

[3] Wang D, Duke R, Chan RP, Campbell JP (2019). Retinopathy of Prematurity in Africa: A Systematic Review. Ophthalmic Epidemiol.

[4] Khorshidifar M, Nikkhah H, Ramezani A, Entezari M, Daftarian N, Norouzi H (2019). Incidence and risk factors of retinopathy of prematurity and utility of the national screening criteria in a tertiary center in Iran. Int J Ophthalmol.

[5] Chiang MF, Quinn GE, Fielder AR, Ostmo SR, Paul Chan RV, Berrocal A (2021). International Classification of Retinopathy of Prematurity, Third Edition. Ophthalmology.

[6] Löfqvist C, Hansen-Pupp I, Andersson E, Holm K, Smith LE, Ley D (2009). Validation of a new retinopathy of prematurity screening method monitoring longitudinal postnatal weight and insulinlike growth factor I. Arch Ophthalmol.

[7] Kadir NA, Ahmad SS, Ghani SA, Bastion MLC (2019). Validation of the WINROP screening algorithm among preterm infants in East Malaysia. Asian J Ophthalmol.

[8] Lim DZ, Oo KT, Tai ELM, Shatriah I (2020). Efficacy of WINROP as a Screening Tool for Retinopathy of Prematurity in the East Coast of Malaysia. Clin Ophthalmol.

[9] Ahmed I, Aclimandos W, Azad N, Zaheer N, Barry JS, Ambulkar H, et al. The Postnatal Growth and Retinopathy of Prematurity Model: A Multi-institutional Validation Study. Ophthalmic Epidemiol. 2022;29:296–301. 10.1080/09286586.2021.1939885 34139931

[10] Yabas Kiziloglu O, Coskun Y, Akman I (2020). Assessment of the GROP study criteria for predicting retinopathy of prematurity: results from a tertiary centre in Turkey. Int Ophthalmol.

[11] Berrocal AM, Fan KC, Al-Khersan H, Negron CI, Murray T (2021). Retinopathy of Prematurity: Advances in the Screening and Treatment of Retinopathy of Prematurity Using a Single Center Approach. Am J Ophthalmol.

[12] Gurwin J, Tomlinson LA, Quinn GE, Ying GS, Baumritter A, Binenbaum G (2017). A Tiered Approach to Retinopathy of Prematurity Screening (TARP) using a weight gain predictive model and a telemedicine system. JAMA Ophthalmol.

[13] Sute SS, Jain S, Chawla D, Narang S (2021). Use of an online screening algorithm - Weight, Insulin-derived growth factor 1, Neonatal Retinopathy of Prematurity (WINROP) for predicting retinopathy of prematurity in Indian preterm babies. Indian J Ophthalmol.

[14] Hellström A, Hård AL, Engström E, Niklasson A, Andersson E, Smith L (2009). Early weight gain predicts retinopathy in preterm infants: new simple, efficient approach to screening. Pediatrics.

[15] Hård AL, Löfqvist C, Fortes Filho JB, Procianoy RS, Smith L, Hellström A (2010). Predicting proliferative retinopathy in a Brazillian population of preterm infants with the screening algorithm WINROP. Arch Ophthalmol.

[16] Wu C, Löfqvist C, Smith LE, VanderVeen DK, Hellström A, the WINROP Consortium. (2012). Importance of postnatal weight gain for normal retinal angiogenesis in very preterm infants: a multicenter study analyzing weight velocity deviations for the prediction of retinopathy of prematurity. Arch Ophthalmol.

[17] Zepeda-Romero LC, Hård AL, Gomez-Ruiz LM, Gutierrez-Padilla JA, Angulo-Castellanos E, Barrera-de-Leon JC (2012). Prediction of retinopathy of prematurity using the screening algorithm WINROP in a Mexican population of preterm infants. Arch Opthalmol.

[18] Ko CH, Kuo HK, Chen CC, Chen FS, Chen YH, Huang HC (2015). Using WINROP as an adjuvant screening tool for retinopathy of prematurity in southern Taiwan. Am J Perinatol.

[19] Piermarocchi S, Bini S, Martini F, Berton M, Lavini A, Gusson E (2017). Predictive algorithms for early detection of retinopathy of prematurity. Arch Ophthalmol.

[20] Kocak N, Niyaz L, Ariturk N (2016). Prediction of severe retinopathy of prematurity using the screening algorithm WINROP in preterm infants. JAAPOS.

[21] Timkovic J, Pokryvkova M, Janurova K, Barinova D, Polackova R, Masek P (2017). Evaluation of the WinROP system for identifying retinopathy of prematurity in Czech preterm infants. Biomed Pap Med Fac Univ Palacky Olomouc Czech Repub.

[22] Jung JL, Wagner BD, McCourt EA, Palestine AG, Cerda A, Cao JH (2017). Validation of WINROP for detecting retinopathy of prematurity in a North American cohort of preterm infants. J AAPOS.

[23] Sanghi G, Narang A, Narula S, Dogra MR (2018). WINROP algorithm for prediction of sight threatening retinopathy of prematurity: Initial experience in Indian preterm infants. Indian J Ophthalmol.

[24] Ueda K, Miki A, Nakai S, Yanagisawa S, Nomura K, Nakamura M (2020). Prediction of severe retinopathy of prematurity using the weight gain, insulin-like growth factor 1, and neonatal retinopathy of prematurity algorithm in a Japanese population of preterm infants. Jpn J Ophthalmol.

[25] Raffa LH, Alessa SK, Alamri AS, Malaikah RH (2020). Prediction of retinopathy of prematurity using the screening algorithm WINROP in a Saudi cohort of preterm infants. Saudi Med J.

[26] Chaves-Samaniego MJ, Gómez Cabrera C, Chaves-Samaniego MC, Escudero Gómez J, García Campos JM, Muñoz Hoyos A (2020). Multicenter validation study of the WINROP algorithm as a method for detecting retinopathy of prematurity. J Matern-Fetal Neonatal Med.

[27] Bai YC, Wu R, Chen SZ, Wei SY, Chen HJ, Chen YC (2021). Efficacy of the WINROP algorithm for retinopathy of prematurity screening in Southern China. Int J Ophthalmol.

[28] Desai S, Athikarisamy SE, Lundgren P, Simmer K, Lam GC (2021). Validation of WINROP (online prediction model) to identify severe retinopathy of prematurity (ROP) in an Australian preterm population: a retrospective study. Eye.

[29] Fernández-Ramón R, Follana-Neira I, Ruiz-Sancho MD (2021). Validation of WINROP algorithm as a screening tool for retinopathy of prematurity in a northern Spanish cohort. Int J Retin.

[30] Almeida AC, Borrego LM, Brízido M, de Figueiredo MB, Teixeira F, Coelho C (2022). DIGIROP efficacy for detecting treatment-requiring retinopathy of prematurity in a Portuguese cohort. Eye.

[31] Athikarisamy S, Desai S, Patole S, Rao S, Simmer K, Lam GC (2021). The Use of Postnatal Weight Gain Algorithms to Predict Severe or Type 1 Retinopathy of Prematurity: A Systematic Review and Meta-analysis. JAMA Netw Open.

